# Antibiotic Targets in Gonococcal Cell Wall Metabolism

**DOI:** 10.3390/antibiotics7030064

**Published:** 2018-07-21

**Authors:** Krizia M. Pérez Medina, Joseph P. Dillard

**Affiliations:** Department of Medical Microbiology and Immunology, University of Wisconsin-Madison, Madison, WI 53706, USA

**Keywords:** peptidoglycan, *Neisseria gonorrhoeae*, lytic transglycosylase

## Abstract

The peptidoglycan cell wall that encloses the bacterial cell and provides structural support and protection is remodeled by multiple enzymes that synthesize and cleave the polymer during growth. This essential and dynamic structure has been targeted by multiple antibiotics to treat gonococcal infections. Up until now, antibiotics have been used against the biosynthetic machinery and the therapeutic potential of inhibiting enzymatic activities involved in peptidoglycan breakdown has not been explored. Given the major antibiotic resistance problems we currently face, it is crucial to identify other possible targets that are key to maintaining cell integrity and contribute to disease development. This article reviews peptidoglycan as an antibiotic target, how *N. gonorrhoeae* has developed resistance to currently available antibiotics, and the potential of continuing to target this essential structure to combat gonococcal infections by attacking alternative enzymatic activities involved in cell wall modification and metabolism.

## 1. Introduction

*Neisseria gonorrhoeae* (gonococcus, GC) is the organism responsible for the sexually transmitted disease gonorrhea. Gonococcal infections cause inflammation that normally manifests as cervicitis in women and urethritis in men. Lack of treatment can lead to infection ascending to the upper part of the reproductive system in women and complications such as pelvic inflammatory disease (PID), chronic pelvic pain, and ectopic pregnancy. A wide variety of antibiotics has been used to treat gonorrhea over the years, but the emergence of resistance has presented a major problem. *N. gonorrhoeae* has shown the ability to develop resistance to all designed antibiotics and the large number of cases of gonococcal infections that occur every year has led the CDC to catalog GC as an urgent threat to public health [1,2,3,4,5,6]. Problems with antibiotic resistance have become increasingly concerning because of the appearance of highly resistant strains in several countries and reports of treatment failures. Highly resistant strains have been identified in Japan, Australia, France, Spain, and the United States (USA) [7,8,9,10,11]. Among the various families of antibiotics implemented, beta-lactam antibiotics targeting the bacterial cell wall have been the most effective for treating gonococcal infections due in part to their longer period of efficacy relative to other antibiotics [6,12]. The expanded spectrum cephalosporins (ESCs) cefixime and ceftriaxone have been the last resorts for monotherapy, but GC strains with decreased susceptibility to these antibiotics and failure of treatment have been recently reported [8,10,11,13,14,15,16,17,18,19]. Widespread antibiotic resistance in *N. gonorrhoeae* in combination with the lack of progress in developing new treatments is threatening our ability to combat gonococcal infections.

The purpose of this article is to review the mechanisms employed by GC to circumvent the activity of currently available antibiotics that target peptidoglycan. We explore the potential of developing new therapeutics that continue to act on this effective target by blocking enzymatic activities involved in modification and metabolism of this essential cellular structure.

## 2. Antibiotic Resistance Related to Peptidoglycan

The peptidoglycan (PG) cell wall has been the target of multiple antibiotics due to its essential role in providing the cell structural support and protection against osmotic stress. PG is a linear polymer comprised of repeating disaccharide units of *N*-acetylmuramic acid (MurNAc) β-1,4 linked to *N*-acetylglucosamine (GlcNAc) with peptide stems attached to each MurNAc residue. PG transpeptidases crosslink peptide stems from adjacent strands and give the PG layer a mesh-like structure. β-lactam antibiotics mimic the substrate of PG transpeptidases, which prevents it from binding to the active site and PG from being cross-linked [20]. *N. gonorrhoeae* has two essential high molecular mass penicillin binding proteins (PBPs) that contain transpeptidase domains and are the main targets of these antibiotics. These biosynthetic enzymes are the class A PBP1 and class B PBP2.

Comparisons between clinical isolates displaying a reduced susceptibility to antibiotics and sensitive strains has allowed for the identification of various factors that contribute to beta-lactam resistance. Multiple studies have shown that alterations in *penA*, which is the gene encoding PBP2, are among these important factors [21,22]. Alignment of *penA* sequences from GC strains recovered from gonococcal infections show the presence of mutations in several positions [23,24]. Given that multiple factors can influence antibiotic resistance, some of the most common *penA* mutations observed have been introduced into sensitive GC strains to assess the specific contribution of alterations in this gene. Most GC strains with reduced susceptibility to β-lactam antibiotics have a mosaic *penA* with up to 60 amino acid changes. This mosaic structure appears to be the result of *N. gonorrhoeae* acquiring portions of *penA* from Neisseria commensals since segments in the sequence have been shown to be identical or highly similar to species such as *N. sicca*, *N. flavescens,* and *N. cinerea* [21,25,26,27]. Introduction of mosaic *penA* alleles from these strains confers resistance to otherwise sensitive strains. Some studies have attempted to identify the specific mutations within the mosaic structure that are responsible for this resistance by introducing individual mutations into sensitive strains or reverting mutated residues to wild-type (WT) in resistant GC. Results from these studies show that substitutions in the C terminal region between residues 500–580 have the biggest impact with regard to antibiotics, which is not surprising given that the targeted transpeptidase domain is encoded in this portion of the sequence [24,28,29]. Epistatic mutations such as I312M, V316T, and G545S do not appear to have an impact when introduced into sensitive GC strains but do significantly affect the resistance to antibiotics when reverted to WT in resistant GC, which were also identified [28]. A similar approach was used to determine which of the additional mutations found in a high level resistant strain are responsible for the transition from intermediate resistance. Mutations A311V, T316P, and T483S were found to account for the increase in antibiotic resistance in strain H041 isolated from Japan [30]. Substitutions in these residues have been observed in other highly resistant GC strains F89 and A886 isolated from France and Australia, respectively [31].

Not all GC strains with reduced susceptibility to antibiotics have a mosaic *penA*. Some important non-mosaic mutations have been identified. The insertion of an aspartic acid residue between residues 345 and 346 was shown to play an important role in the decreased susceptibility to penicillin [32]. More recently, the substitution of the alanine in position 501 was determined to be particularly important in resistance to the expanded spectrum cephalosporin cefixime without requiring additional mutations [26,33].

The mechanism by which mutations observed in *penA* result in the resistance to antibiotics is not well understood. Limited structural information has been obtained for PBP2 from resistant GC strains to evaluate how different mutations affect the protein structure. Crystal structures from *N. gonorrhoeae* CDC84-060418, 6140CT (ommiting Asp345 insertion), and 6140CT-A501T (substitution A501T inserted) show only subtle differences when compared to WT [33,34]. The lack of drastic changes in the structure is not surprising given that the enzyme is essential and must, therefore, remain active against PG substrates. Unfortunately, structures have only been solved for PBP2 containing four to six mutations in the C-terminal portion. A mosaic PBP2, which might display bigger structural changes, has not been successfully crystallized. The C-terminal transpeptidase domain comprises two subdomains including an α subdomain and an α/β subdomain with the active site cleft located between them [20]. The α/β subdomain that forms the lower face of the active site contains a structurally disordered region within its antiparallel β-strands core. The most notable change in the solved structures of mutated PBP2 is the ordering of the *B*3-*B*4 loop located in this region [20,33,34]. This more ordered loop appears to increase rigidity in the vicinity of the active site, which could interfere with the ability of the enzyme to undergo conformational changes. A slight shift that brings *B*3 closer to the active site could also contribute to the decreased susceptibility by a difficult access to the active site.

Predictions have been made based on the available structures regarding the effect that other known mutations could have in the structure and the response to antibiotics. Most of the mutations consistently observed in resistant strains are found in proximity to the active site where some of them can participate in hydrogen bonds with residues from conserved motifs [28,30,32,33,34]. Substitutions in these residues could perturb these interactions and even create new contacts that interfere with the enzyme’s affinity for the antibiotic or de-stabilize the transition state, which lowers the acylation rate.

Other aspects besides structural changes have been evaluated in order to better understand the mechanism behind antibiotic resistance. Mutations that increase minimum inhibitory concentrations (MIC) have been further studied to assess their effect on the acylation rate and protein stability. Interaction of the enzyme with the antibiotic leads to formation of an intermediate acyl-enzyme complex and the kinetics of this reaction can be measured using radiolabeled penicillin. The acylation rate constants k_2_/K_s_ serve as a direct measure of enzyme inactivation efficiency, which depends on the rapid formation and accumulation of the acyl-enzyme. Calculation of these constants for multiple PBP2 variants has shown the varying degree to which different mutations affect acyl-enzyme kinetics and how changes in acylation rate differ depending on the antibiotic. Changes in the acylation rate have been measured for penicillin and other important antibiotics like the ESCs cefixime and ceftriaxone. Results mostly correlate with the reported changes in MIC. These comparative studies showcase the contribution of specific residues to the resistance of a particular antibiotic. For example, experiments that address the importance of A501 substitutions in PBP2 for antibiotic response led to the observation that adding substitutions results in an increase in the acylation rate of penicillin. However, it has an opposite effect with cefixime [33]. A similar approach has been taken with high level resistance strains to evaluate the impact that additional mutations have on the kinetics of enzyme-antibiotic interaction. Results from these experiments confirmed the previously mentioned key role of substitutions A311V, V316P, and T483S in the transition from intermediate to high level resistance in strain H041 [30]. A final aspect that has been investigated for some mutations is how they affect thermal stability. This analysis has been accomplished by collecting thermal stability curves for wild-type and mutated PBP2. Results show that mutations affecting PBP2 lead to a lower melting temperature, which indicates a destabilizing effect of the amino acid substitutions on the protein [33,34].

As previously mentioned, *N. gonorrhoeae* encodes another PBP that contains a transpeptidase domain and could be targeted by β-lactam antibiotics, PBP1. PBP2 has been the main focus in antibiotic resistance studies because PBP1 has a lower affinity for developed antibiotics and, unlike *penA*, major changes in the sequence of *ponA*, which is the gene encoding PBP1, have not been observed in resistant GC strains [35,36]. However, one point mutation has been consistently detected in gonococci that are highly resistant to penicillin, L421P [21,37,38,39]. This one mutation has been shown to reduce affinity to penicillin and contribute to the transition from intermediate resistance when introduced in strains containing other antibiotic resistance determinants. The combination of mutations in both *penA* and *ponA* results in GC strains with high level antibiotic resistance.

## 3. Peptidoglycan-Degrading Enzymes as Potential Antibiotic Targets

PG is a dynamic structure that is constantly being remodeled to accommodate cell growth and division. Multiple enzymes responsible for cleaving PG and synthesizing PG are involved in this process [40,41,42]. Up until now, antibiotics used against the process of cell wall remodeling have targeted enzymes involved in the synthesis of the polymer instead of the breakdown. Despite no single enzyme being essential for cell survival, PG-degrading enzymes carry out required functions in the cell. Thus, researchers should take a closer look at these enzymes in the search for potential new targets for the treatment of gonococcal infections.

**Figure 1:**
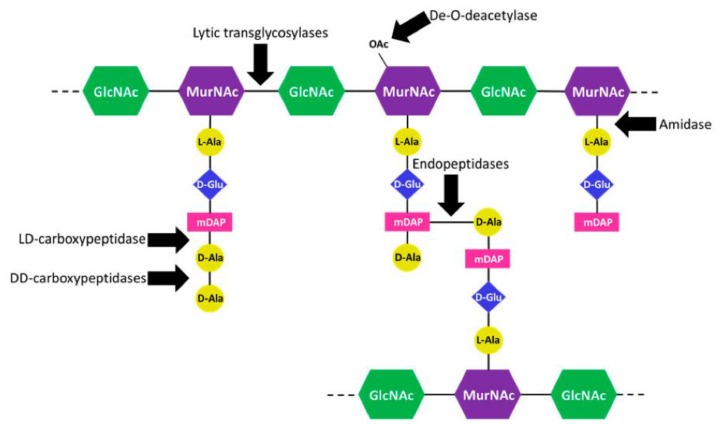
Cleavage site of gonococcal peptidoglycanases. Lytic transglycosylases cleave the β-1,-4 link between the *N*-acetylmuramic acid (MurNAc) and *N*-acetylglucosamine (GlcNAc) sugar moieties. DD-carboxypeptidases and a LD-carboxypeptidase shorten the peptide stem. The LD-carboxypeptidase can only act on four amino acid peptide stems. An *N*-acetylmuramyl L-alanine amidase cleaves the stem off the MurNAc residue. Endopeptidases cut the crosslinks between peptide chains from adjacent PG strands. A PG de-*O*-deacetylase removes the *O*-acetyl group from MurNAc. Without de-acetylation, the lytic trans-glycosylases cannot cleave the MurNAc bond.

Several classes of peptidoglycanases act on PG to cleave the different bonds that hold the mesh-like structure together (Figure 1). Carboxypeptidases shorten the peptide stem attached to the MurNAc residue while amidases are responsible for cleaving the stem off the sugar moiety. Endopeptidases cleave the crosslinks between peptide stems from adjacent PG strands generated by PG transpeptidases, and lytic transglycosylases separate the GlcNAc and MurNAc sugar moieties that are brought together by PG transglycosylases. The activity of all these different enzymes during growth leads to the production of diverse PG fragments that can be recycled back into the cell or can be released to the environment. Relative to other organisms, gonococcus releases a large percentage of the produced fragments instead of recycling them [43,44,45,46].

PG degradation enzymes have been studied in *N. gonorrhoeae* because they are required for cell septation and separation as well as because the PG fragments generated are toxic to the host tissue. PG fragments released during bacterial growth have been shown to replicate the pathology observed in patients during gonococcal infections. Using human fallopian tubes in organ culture, Melly, McGee, and Rosenthal demonstrated that exposure to PG fragments leads to the sloughing of ciliated cells [47]. These observations sparked the interest of researchers and prompted further research to elucidate the mechanism behind the damage caused by the fragments. Recently, it was discovered that released (muro)peptides can be sensed by a pair of intracellular pattern recognition molecules known as NOD receptors in multiple bacterial infections [47,48,49,50]. Recognition of PG fragments by receptors NOD1 and NOD2 induces expression of inflammatory cytokines associated with tissue damage such as IL-1, IL-6, IL-8, and TNF-α [51,52,53,54,55].

It was discovered that human NOD1 (hNOD1) receptors, which are expressed by many different cell types, recognize PG fragments with a terminal mDAP in the peptide stem [54,56,57]. However, hNOD2 receptors expressed by a limited set of cell types bind to muramyl dipeptide, whole sacculi, or monomeric PG fragments with a reducing end. Gonococcus normally releases PG dimers, monomers, free peptides, and free disaccharide [58]. Of special interest are tripeptides and tripeptide monomers because they are the most abundant PG fragments released by GC and they have been shown to act as hNOD1 agonists. 

The inflammatory response to gonococcal infection appears to be responsible for disease. For this reason, NOD1 activation represents an important pathogenic function in gonococcal infections. Production of the tripeptide monomer requires lytic transglycosylases (LT) to cleave the glycan backbone while tripeptide production requires amidase activity to cleave the peptide stem off the sugars. Even though *N. gonorrhoeae* encodes for seven to nine lytic transglycosylases that can cleave the bond between MurNAc and GlcNAc, only two of them, LtgA and LtgD, are involved in the production of monomers that get released [59,60,61]. On the other hand, GC only has one periplasmic *N*-acetylmuramyl L-alanine amidase, AmiC. Both AmiC and the LTs LtgA and LtgD were found to contribute to hNOD1 activation [62]. Approximately 70% to 80% of the peptide stems in the gonococcal sacculus are tetrapeptides [46]. This is in stark contrast to the composition of the PG fragments released during growth, which mainly contain a tripeptide stem. The differences in the structural composition of PG in the sacculus and liberated fragments point to the requirement of carboxypeptidase activity to shorten the peptide stem in order to produce both hNOD1 agonists. The identity of the L,D-carboxypeptidase responsible for converting tetrapeptides into tripeptides was revealed to be LdcA [63]. Inactivation of LdcA leads to a shift in the proportion of PG monomers released relative to the wild-type from mostly tripeptide monomer to almost completely tetrapeptide monomer. More importantly, elimination of LdcA activity significantly decreases not only NOD1 but also NOD2 activation and attenuates the host inflammatory response to GC. GC has approximately 40% of its PG crosslinked, which suggested that endopeptidases could affect the amount of hNOD1 agonists released [64]. Enzymes PBP3 and PBP4 encoded by *N. gonorrhoeae* have been shown to have endopeptidase activity [65,66]. A strain with both these enzymes inactivated displays a very large decrease in PG monomers released and the complete elimination of free peptides. These changes in the PG fragments produced impaired GC from activating the NOD1 pathway. Endopeptidases playing such a key role in hNOD1 agonist production was surprising given that more than half of the PG in the sacculus is uncrosslinked. 

The role of peptidoglycanases in the creation of bacterial products that influence the host response is not the only aspect that points to PG degrading enzymes as potential targets for treatment. Previous work has shown that some of these enzymes are particularly critical for proper daughter cell separation. Absence of the lytic transglycosylase LtgC or the amidase AmiC results in bacterial cell aggregates sharing a single cell wall and outer membrane [67,68]. It was later shown that the absence of another protein that functions as an amidase activator, NlpD, leads to the same deficiency in cell separation [69]. Studies conducted with other organisms have shown that infection with mutants that have aberrant cell separation does not lead to disease development. The inability of an *N. meningitidis* strain defective in cell separation to establish bacteremia and attenuation of a *Y. pestis nlpD* mutant suggest that this process may be a good target for new therapeutics [70,71]. It has also been shown that these mutants have increased permeability to antibiotics, which means that interfering with the activity of these enzymes could potentially improve the efficacy of currently available antibiotics.

During cell wall remodeling, a balance between PG breakdown and synthesis is required to ensure cell integrity despite all the enzymatic activities involved in the process. Preserving cell integrity by maintaining this delicate balance and coordinating the various enzymatic activities is crucial for the bacterium to retain protection from host immune responses. Recent evidence comes from studies of gonococci interacting with neutrophils. A significant portion of *N. gonorrhoeae* is able to resist and survive the antimicrobial arsenal of neutrophils [72,73,74]. Studies showed that mutation of lytic transglycosylases *ltgA* and *ltgD* leads to a decrease in survival [75]. Decreased resistance in this double mutant was shown to be independent of PG monomer release because the addition of the monomer did not restore susceptibility. It was discovered that GC succumbs to neutrophils when the activity of these lytic transglycosylases is impaired due to increased susceptibility to lysozymes and elastase deployed by neutrophils. Lack of LtgA and LtgD activity compromises the integrity of the membrane barrier and allows for the access of these neutrophil antimicrobials to bacterial cells that would otherwise be protected by preventing access of these molecules. These results suggest that disturbing the integrity of the barrier that represents the bacterium’s first line of defense by blocking the activity of PG breakdown enzymes could assist the host immune system in the clearance of infection. It remains to be determined if inhibiting other PG breakdown enzymatic activities could also lead to an increased susceptibility to cells employed by the host immune system during infection to kill the pathogen. Another aspect that has not been assessed is the effect that impeding these enzymes could have in clearance by other immune cells. GC has also been shown to survive and replicate inside macrophages [76,77,78]. Identifying methods to prevent *N. gonorrhoeae* from evading killing by these immune cells is of great interest because this could be a mechanism that contributes to the persistence and resurfacing of gonococcal infections. 

Another benefit has been reported to add to the therapeutic potential of targeting lytic transglycosylases. Preventing their activity does not only result in more susceptibility to the host immune system but also to β-lactam antibiotics. The lytic transglycosylase inhibitor bulgecin A provides an example of this phenomenon. Combination of the inhibitor with β-lactam antibiotics has been shown to decrease MIC of β-lactams such as penicillin G, amoxicillin, and cefotaxime [79]. Efficacy improvement of β-lactams by bulgecin A has also been observed in other organisms like *Helicobacter pylori*, *E. coli*, and *Neisseria meningitidis* [79,80,81]. These results demonstrate the therapeutic potential of lytic transglycosylase inhibitors due to their synergism with β-lactams. Experiments show that dual therapy with these inhibitors could restore the susceptibility of resistant GC strains to β-lactam antibiotics already developed. LT inhibitors like bulgecin A and NAG-thiazoline could provide insight for the design of therapeutics that could be used in a dual-therapy regimen for gonococcal infections [79,80,82].

As previously mentioned, PG is modified by several enzymes. Besides those in charge of breaking down and synthesizing the polymer during growth, there are other enzymes carrying out another important modification to PG composition known as PG *O*-acetylation. This modification is known to affect susceptibility to lysozyme degradation and virulence of some pathogens [83,84,85,86,87,88,89,90]. Studies have been done to understand the role this process and the enzymes involved play in *Neisseria* physiology and virulence. Results from these studies showed that preventing de-*O*-acetylation of PG by deleting the acetyl PG esterase 1 (Ape1) that fulfills this function leads to a drastic defect in virulence. Using a murine model of sepsis, it was shown that infection with an *N. meningitidis* strain lacking Ape1 leads to a significantly lower level of bacteremia [91]. De-*O*-acetylation appears to be an important process required for the invasiveness of the pathogen. Given the attenuation observed in *N. meningitidis*, Ape1 appears to be yet another possible target for new therapeutics to prevent disease and promote elimination by the immune system. Further work must be done to determine if inhibition of de-*O*-acetylation has a similar effect in the ability of *N. gonorrhoeae* to persist and cause disease.

## 4. Protein-Protein Interactions Involved in Cell Wall Remodeling as Potential Antibiotic Targets

It is not completely understood how bacteria coordinate the activity of enzymes cleaving PG and synthesizing it to ensure the integrity of the PG layer during growth and remodeling. A tight control must be in place to avoid autolysis or defects in cell separation. It has been proposed that the mechanism behind this regulation is the formation of multi-enzymatic complexes that allow for the coordination of the various activities [92,93,94,95]. Studying the presence and composition of these complexes is important for understanding how enzymes required for survival and others that appear to be important for disease development exert their activity. Along with shedding a light into how the cellular process of cell wall remodeling works, results from these studies could provide insight into alternative mechanisms that could be employed to continue to target the essential biosynthetic machinery and/or the yet-to-be targeted breakdown enzymes. There are peptidoglycanases encoded by GC that might be critical and affect survival, the production of toxic PG fragments, or proper daughter cell separation by regulating the activity of the enzymes directly involved in these processes through protein-protein interactions. Interfering with the formation of enzymatic complexes by peptidoglycanases represents another possible approach for new treatments. 

Multiple studies have been conducted to identify protein-protein interactions to support the multi-enzymatic complex model. Most of these studies have focused on the proteins that are recruited to the division site early during cell division to constrict the cells and start the process of separation into two daughter cells, which has led to the elucidation of the divisome [96,97,98]. Other studies have been designed to identify interacting partners of enzymes involved in the release of toxic PG fragments, but they have only focused on the relationship between lytic transglycosylases and penicillin binding proteins (PBP). These enzymes have been at the center of many studies given their requirement for PG monomer release and the ability of monomers to cause tissue damage. 

Research into the formation of multi-enzymatic complexes by peptidoglycanases has led to the detection of multiple interacting partners among not only enzymes responsible for cleaving PG but also among those involved in its synthesis. Interactions have been seen between enzymes responsible for cleaving PG at different sites. For example, an interaction between the lytic transglycosylase Slt70 and the endopeptidase PBP7 was detected in *Escherichia coli* [99]. Enzymes involved in assembling the polymer also appear to interact among themselves [94,95,100]. Using multiple techniques, the bifunctional transglycosylase-transpeptidase murein synthase PBP1B was shown to interact with the transpeptidase PBP3 in *E. coli* [100]. Not all the interactions that have been observed occur between enzymes involved in the same process of PG synthesis or breakdown. Some interactions connect these opposing functions with representatives from the contrasting groups coming together. Examples of interactions between synthetic and breakdown enzymes have been observed in various organisms. The interactions between lytic transglycosylases and the PBPs responsible for transglycosylation and transpeptidation have been detected in organisms such as *E. coli*, *Pseudomonas aeruginosa*, and *N. meningitidis* [101,102,103,104]. 

Some of the elucidated interactions are known to be vital for the activity of the enzymes because they are responsible for recruitment to the site of action. This function has been confirmed for the synthetic pair PBP1B and PBP3 in *E. coli* and for the PG amidase AmiC and its activator. PBP1B requires its interaction with PBP3 to localize to the division site [100]. In the case of PG amidases and their activators, there appears to be variation in the requirement of the activator for amidase localization among organisms. PG amidase AmiC in *E. coli* can localize to the division site independently of its activator NlpD while the homolog in *Vibrio cholerae* known as AmiB requires the presence of one of its activators NlpD or EnvC to localize [105,106]. This last result highlights the importance of caution when generalizing about protein-protein interactions and their role based on results from another organism.

A major obstacle for studying the interactions among peptidoglycanases has been the high level of redundancy in the organisms studied so far. This has also raised concerns regarding the potential of exploiting discovered interactions for the development of new treatments. However, contrary to other organisms, *N. gonorrhoeae* has a relatively small number of peptidoglycanases with fewer enzymes of each class and less redundancy of function. Further research is needed to address questions regarding the importance of these enzymes and enzymatic complexes in proper cell separation, in the production of toxic PG fragments, and in host tissue damage. The lack of redundancy observed in GC suggests that answers to these questions can lead to the discovery of new targets for the development of future treatments that affect cell integrity and impair the ability of *N. gonorrhoeae* to cause disease.

## 5. Discussion

Antibiotic resistance is making treatment of gonococcal infections increasingly difficult. The efficacy of antibiotics currently used for treatment is declining and, without new therapeutics in sight, we currently face the threat of untreatable gonorrhea [107]. Multiple antibiotics used to combat *N. gonorrhoeae* target PG by blocking the activity of the essential biosynthetic enzymes PBP1 and PBP2. There are PG breakdown enzymes whose activity is also important for the bacterium to maintain the integrity of the cell during growth and that represent unexplored avenues for treatment. As discussed in this paper, the activity of these enzymes causes the production and release of small PG fragments including some which can be detected by intracellular pattern recognition receptors NOD1 and NOD2. Therefore, these enzymes should be considered not just as virulence factors but also as immunomodulatory. Their inhibition would lead to a different immune response and possibly to better clearance of the bacteria. Special attention should be given to the gonococcal carboxypeptidases and endopeptidases required for the production of both hNOD1 agonists given that results suggest that the release of both the tripeptide monomer and the tripeptide must be affected to impair activation of the NOD1 pathway. One of the reasons that antibiotics have not been designed to target the PG breakdown enzymes is because they are not essential for growth. Even though these enzymes are not individually essential for growth under laboratory conditions, it is unknown if they are required for the bacterium to survive in the host in the context of infection. The reason for GC releasing PG fragments that assist the host in detecting the pathogen is not understood but results suggest that they fulfill an important function because *N. gonorrhoeae* has modifications in its recycling machinery that cause the changes in the proportion of recycled and released PG fragments [108]. The importance of some PG breakdown enzymes during infection has been demonstrated in *N. meningitidis* where a lytic transglycosylase and an endopeptidase mutant were unable to cause disease in the infant rat model [67,71,109]. Addressing the importance of peptidoglycanases for gonococcal infections has been difficult because GC is an obligate human pathogen and animal models do not replicate most aspects of human disease.

Organisms that have been used to study the enzymes involved in cell wall remodeling like *E. coli* show a lot of redundancy. This redundancy makes it more difficult to target the PG degrading enzymes because the bacterium can make up for the loss of activity of individual enzymes. Unlike these organisms, *N. gonorrhoeae* appears to have a more streamlined machinery with fewer members from the different classes, which highlights the potential of targeting the process of cell wall remodeling by blocking the activity of additional enzymes involved in the process. LdcA, PBP3, and PBP4 appear to be good candidates for future therapeutics given their requirement for producing toxic PG fragments. Studies have shown an inability of GC to activate the NOD1 pathway and a decrease in NOD2 activation when these peptidoglycanases are absent [63]. Further work is needed to better understand the importance of released PG fragments and the activation of these pathways for infection and disease development. 

Blocking activity of PG breakdown enzymes like lytic transglycosylases compromises the integrity of the bacterium’s membrane barrier and facilitates the access of antimicrobials [75,79]. This disturbance has demonstrated therapeutic potential by restoring the efficacy of β-lactams in resistant GC strains. Alterations in the PG composition appear to influence the pathogen’s ability to persist in the host and cause disease, which makes the enzymes involved in PG modification and metabolism potential targets for new therapeutics. The enzyme Ape1 responsible for de-*O*-acetylation of PG represents a great example given that deficiency in this process has been shown to impair the mutants from causing disease in other organisms [91].

There are other PG hydrolases that do not impact the composition or the amount of PG fragments released but are important for proper daughter cell separation. AmiC, LtgC, and NlpD are essential for this process in the gonococcal cell and their absence leads to bacterial growth in clusters with multiple cells sharing an outer membrane [67,68,69]. More research should be done with cell separation mutants to address the impact that this defect has in the susceptibility to the host immune response and to antibiotics.

Peptidoglycanases appear to be regulated by the formation of multi-enzymatic complexes that allow for the coordination of the diverse activities on PG [92,93,94,95]. This represents another largely unexplored path that could be exploited for treatment. The composition of these enzymatic complexes must be elucidated to address their importance for proper activity of the individual enzymes. Enzymes that are not directly and actively involved in processes essential for the survival of the bacterium and essential for disease might turn out to be critical because they control the activity of the enzymes involved. Results from multiple studies showing protein-protein interactions among different classes of peptidoglycanases support the multi-enzymatic complex hypothesis [94,100,101,102,103,104]. Most of the protein-protein interaction studies have not investigated beyond the identification of interacting partners, which means many questions remain unanswered regarding the importance and the role of these interactions for disease.

More research is needed to assess the potential of the enzymes discussed in this paper as targets for future antibiotics. Promising results have been obtained from work with lytic transglycosylases, which show that a lytic transglycosylase inhibitor increases the efficacy of β-lactam antibiotics [79]. It remains to be determined if targeting activity of these and other gonococcal peptidoglycanases could prevent disease or increase the susceptibility of the pathogen to the host immune response and established antibiotics. Work in this area could lead to the design and development of new therapeutics that would effectively combat gonococcal infections as monotherapies or therapeutics that increase the efficacy of currently available antibiotics.
